# Clinical Utility of Optical Genome Mapping for Improved Cytogenomic Analysis of Gliomas

**DOI:** 10.3390/biomedicines12081659

**Published:** 2024-07-25

**Authors:** Harmanpreet Singh, Nikhil S. Sahajpal, Ashis K. Mondal, Stephanie L. Burke, Jaspreet Farmaha, Ahmet Alptekin, Ashutosh Vashisht, Kimya Jones, Vishakha Vashisht, Ravindra Kolhe

**Affiliations:** 1 Department of Pathology, Medical College of Georgia, Augusta University, Augusta, GA 30912, USA; hsingh1@augusta.edu (H.S.); amondal@augusta.edu (A.K.M.); jfarmaha@augusta.edu (J.F.); avashisht@augusta.edu (A.V.); kjones2@augusta.edu (K.J.); vvashisht@augusta.edu (V.V.); 2 Greenwood Genetic Center, Greenwood, SC 29646, USA; nsahajpal@ggc.org; 3 Clinical and Scientific Affairs, Bionano Genomics, San Diego, CA 92121, USA

**Keywords:** optical genome mapping, structural variants, solid tumor, glioma, chromosomal microarray

## Abstract

A glioma is a solid brain tumor which originates in the brain or brain stem area. The diagnosis of gliomas based on standard-of-care (SOC) techniques includes karyotyping, fluorescence in situ hybridization (FISH), and chromosomal microarray (CMA), for detecting the pathogenic variants and chromosomal abnormalities. But these techniques do not reveal the complete picture of genetic complexity, thus requiring an alternative technology for better characterization of these tumors. The present study aimed to evaluate the clinical performance and feasibility of using optical genome mapping (OGM) for chromosomal characterization of gliomas. Herein, we evaluated 10 cases of gliomas that were previously characterized by CMA. OGM analysis showed concordance with the results of CMA in identifying the characterized Structural Variants (SVs) in these cases. More notably, it also revealed additional clinically relevant aberrations, demonstrating a higher resolution and sensitivity. These clinically relevant SVs included cryptic translocation, and SVs which are beyond the detection capabilities of CMA. Our analysis highlights the unique capability of OGM to detect all classes of SVs within a single assay, thereby unveiling clinically significant data with a shorter turnaround time. Adopting this diagnostic tool as a standard of care for solid tumors like gliomas shows potential for improving therapeutic management, potentially leading to more personalized and timely interventions for patients.

## 1. Introduction

Gliomas originating from the glial or supporting cells in the Central Nervous System (CNS), constitute a diverse spectrum of neuroepithelial tumors. According to the latest Surveillance, Epidemiology, and End Results (SEER) report, the incidence rate of brain cancer was 5.9 per 100,000 people in 2021, and in 2024, it is estimated that there will be 25,400 new cases of brain cancer, which constitutes the 1.3% of all new cancer cases in the U.S. [1]. Gliomas constitute the most common form of malignant tumor of the CNS, exhibiting a wide histological spectrum, from benign ependymomal tumors to aggressive and deadly grade IV glioblastoma (GBM) with a median survival rate of 15 months [2,3]. These solid tumors were traditionally classified using histological features only. However, in 2016, the World Health Organization (WHO) introduced molecular markers to classify CNS tumors (CNS WHO 4). This molecular characterization was further reinforced by the latest WHO Classification of Tumors of the CNS (WHO-2021) guidelines, by advocating for integrated diagnosis that combines genetic markers, molecular profiles, and histology for precise sub-typing of these solid tumors [4]. In addition to genetic alterations in isocitrate dehydrogenase (*IDH1/2*) and 1p/19q-codeletion, WHO-2021 guidelines include additional molecular markers such as *H3-3A*, *ATRX*, *CDKN2A/B*, *TERT*, *MGMT,* and *EGFR* to classify gliomas.

While acknowledging the importance of precise molecular aberrations in prognosis determination, treatment response, and personalized therapy, WHO-2021 guidelines also include a NEC (Not Elsewhere Classified) diagnosis for cases where conventional diagnostic testing fails to yield a specific diagnosis [4]. The current cytogenetic analysis of gliomas relies on conventional methods that include karyotyping, fluorescence in situ hybridization (FISH), and chromosomal microarray (CMA). Karyotyping, while proficient in detecting copy number variations (CNVs) and balanced/unbalanced structural variations (SVs), encounters limitations due to its banding resolution (5–10 Mb), necessity for cell culturing, and sensitivity to the technical skills of the technicians [5]. FISH has higher resolution in detecting chromosome abnormalities but is not capable of unraveling novel SV events. CMA has a higher resolution of approximately 50–100 kb for the detection of CNVs; however, it has limited utility in detecting balanced rearrangements or the orientation of duplicated segments, identifying the location of inserted segments, and is unable to detect low-frequency allelic changes. Thus, a more robust technique that overcomes these conventional drawbacks is needed for a more detailed molecular characterization of the gliomas to further refine their sub-typing and hence improve patient outcomes.

Optical genome mapping (OGM), a high-resolution imaging approach that combines optical microscopy with computational algorithms, has recently emerged as a next-generation cytogenomic technology for molecular diagnosis in cancer and non-cancer diseases. With its resolution of 500 bp, OGM helps in the detection of complex rearrangements and different classes of SVs (inversions, translocations, insertions, deletions, duplications) in a single step, providing a detailed picture of the genome in a timely manner [6]. Our recent publications highlight the use of this state-of-art cytogenomic technology in clinical diagnosis of hematological malignancy [7], acute myeloid leukemia [8], and for prenatal genomic analysis [9]. In this study, we present the first use of OGM as a complementary approach for the detection of various cryptic and complex rearrangements and SVs involved in gliomas beyond the limit of detection of the SOC testing.

## 2. Materials and Methods

### 2.1. Sample Collection

This study included 10 deidentified, frozen, glioma brain tumors obtained from the tumor bank at Georgia Cancer Center, Augusta University. These archived, residual biopsy samples were previously characterized using chromosomal microarray for clinical diagnosis purposes at Augusta University Medical Center and were subsequently reanalyzed using OGM as part of the current comparative study. The diagnostic reports, devoid of any patient identifiers, were obtained with the samples.

### 2.2. Optical Genome Mapping: DNA Isolation, Labeling, and Data Collection

DNA Isolation from solid tissue samples: Ultra-high-molecular-weight (UHMW) DNA was isolated from the solid tumor tissue samples using the Bionano Prep SP Tissue and Tumor DNA Isolation kit (Bionano Genomics, San Diego, CA, USA, Cat. No. 80038) per the manufacturer’s protocol. The protocol is described in greater detail in the Bionano support document [10]. The isolated UHMW DNA was labeled and processed for analysis on the Bionano Genomics Saphyr^®^ platform following the manufacturer’s protocols (Bionano Genomics Inc., San Diego, CA, USA). Briefly, the frozen tissue samples were weighed, and approximately 30 mg of brain tissue was used for the DNA isolation. DNA isolation was performed as per the standard protocol optimized for solid tumors. Finally, isolated DNA was eluted in the elution buffer and homogenized overnight at room temperature, followed by quantification using Qubit broad-range double-stranded DNA assay kits (ThermoFisher Scientific, San Francisco, CA, USA).

Direct Label and Staining (DLS): 750 ng of UHMW DNA was labeled using the Bionano Direct Label and Stain (DLS) kit (Bionano Genomics, San Diego, CA, USA, Cat. No. 80005) as per the manufacturer’s protocol for DNA labeling and quantified using Qubit High Sensitivity (HS) double-stranded DNA assay kits. The fluorescently labeled UHMW DNA was then loaded onto flowcells of a Saphyr^®^ chip, electrophoretically linearized within the nanochannel arrays, and imaged using the Bionano Saphyr^®^ instrument.

OGM Variant Calling and Data Analysis: Genome analysis was performed using the rare variant pipeline included in the Bionano Access v.1.7.2/Bionano Solve (v.3.7.2) software (Bionano Genomics, San Diego, CA, USA). Briefly, the DNA molecules from each sample were directly aligned against the reference human genome assembly (hg19), and the SVs were detected based on the differences in the alignment of the labels between the reference assembly and samples. Consensus genome maps were assembled, and final SVs were called, having reported a minimum of five molecules. SVs and CNVs generated by the rare variant pipeline were then annotated and analyzed using known hg19 pan-cancer bed files to determine clinical relevance. Analytical quality control (QC) targets were set to achieve >300× effective coverage of the genome, >70% mapping rate, 13 to 17 label density (labels per 100 kbp), and >220 kbp N50 (of molecules >150 kbp).

### 2.3. Chromosomal Microarray

UHMW DNA was isolated from tumor tissue following manufacturer’s protocol. OGM and CMA were performed using the same DNA to remove tumor/tissue-dependent bias. The genomic DNA (gDNA) for SNP microarray was analyzed using the CytoScan HD assay following the manufacturer’s protocol (ThermoFisher Scientific, Waltham, MA USA) using hg19 reference assembly.

## 3. Results

### 3.1. Quality Control Metrics of the Samples

All 10 samples passed the quality control metrics and achieved the minimum average parameter required to run the rare variant pipeline (RVP) analysis with an average N50 (>150 kb) of 260 kb (±46.2), map rate of 86.4% (±3.7%), label density of 15.1/100 kb (±0.82), and average coverage of 413.8X (±30.5) (Supplementary File Table S1).

### 3.2. OGM Showed Concordance with the Results of CMA

**Figure 1:**
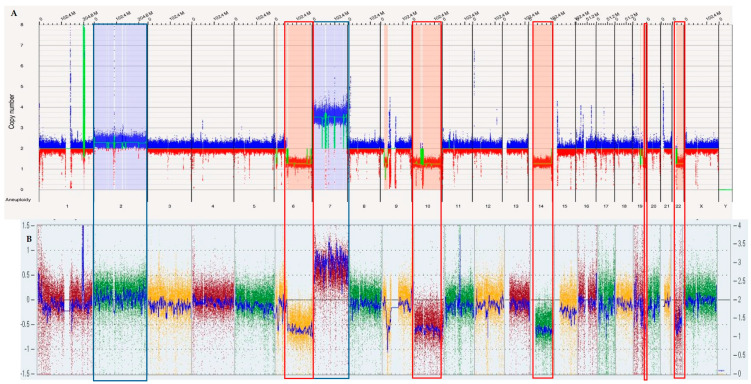
Whole genome view showing the concordance of the copy number variations detected +2, 6q-, +7, -10, -14, 19q-, and -22 by OGM (**A**) and CMA (**B**).

**Figure 2:**
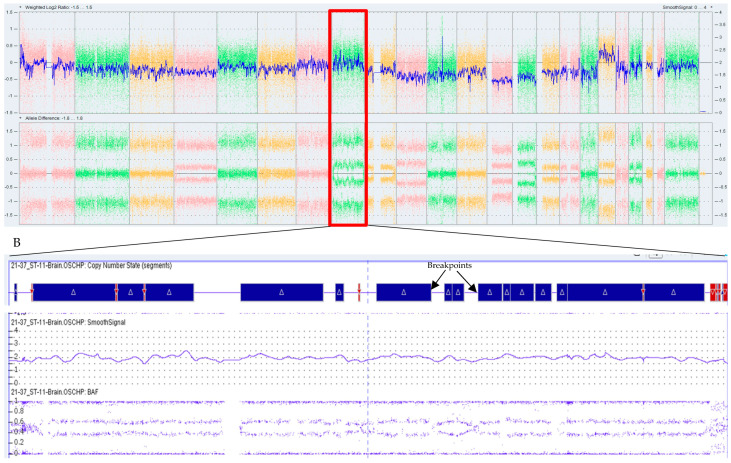
(**A**) The chromothripsis event detected on chromosome 8 in microarray, (**B**) the enlarged view of the same event indicating the various breakpoints by microarray where blue is gain and red is loss of CN.

**Figure 3:**
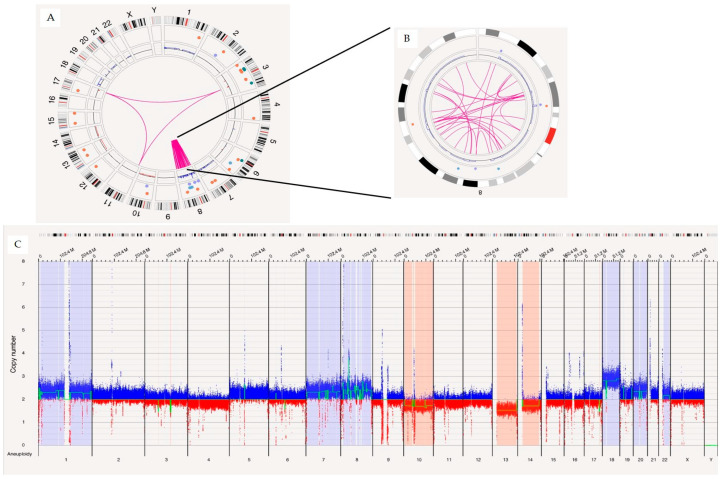
The OGM circus plot (**A**) showing the chromothripsis event observed on chromosome 8 with an enlarged view of chromosome 8 and (**B**) showing the SV events related to the chromothripsis call. On the bottom (**C**) is the OGM whole genome view as a secondary visualization confirming the chromothripsis event in this case, blue color indicates CMV gain segment and red color indicates CNV loss in chromosomes.

OGM demonstrated concordance with CMA results, excluding those results related to the loss of heterozygosity calls. For instance, in one case, CMA identified multiple aneuploidies and CNVs, including +2, 6q−, +7, −10, −14, 19q−, and −22. These SVs were also identified by OGM, as shown in Figure 1, which shows the overlapping copy number variations between OGM and CMA. In another case, CMA identified chromothripsis involving chromosome 8 (Figure 2), with the different breakpoints visible in an enlarged view of the call. OGM detected the same chromothryptic event in chromosome 8 (Figure 3). By enlarging the OGM calls on chromosome 8, various breakpoints and fusion events involved in the chromothripsis were visualized. This finding was further confirmed in the whole genome view, with a visualization of the highlighted segmental changes.

### 3.3. OGM Clearly Defined an SV Event

**Figure 4:**
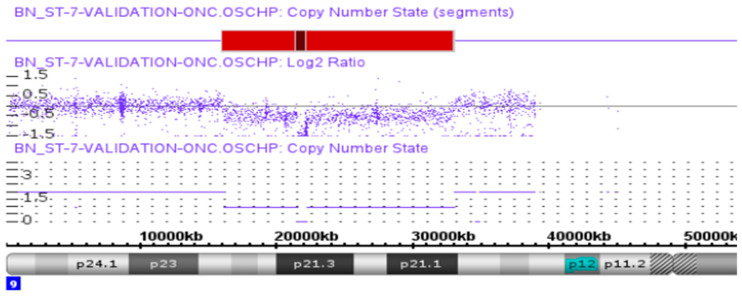
The copy number loss and the nested deletion involving the *CDKN2A* and CDKN*2B* genes identified by CMA analysis in one of the glioma cases. Light red is the CNV event and dark red is the nested deleted present in the CNV loss event which contain the *CDKN2A* and CDKN*2B* genes.

**Figure 5:**
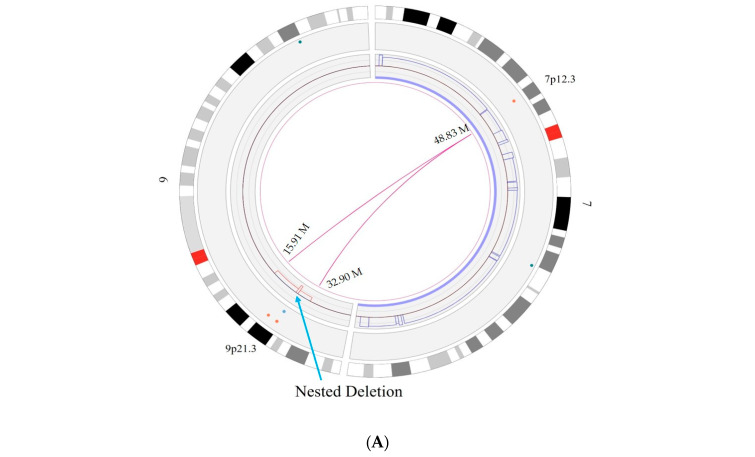

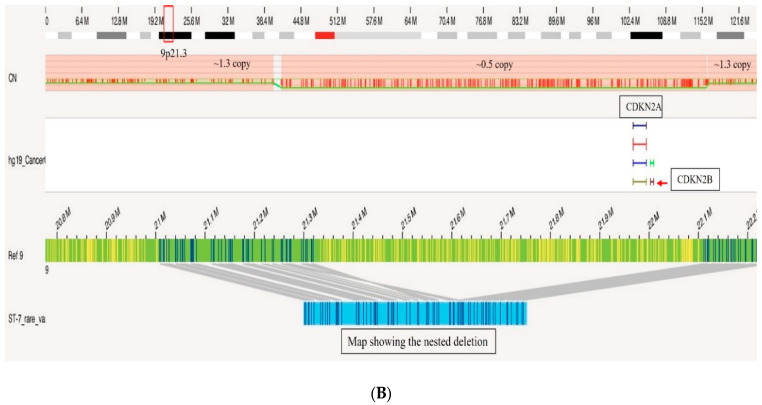
OGM reveals additional details for a glioma case presented in Figure 4. (A) unbalanced translocation event between chromosome 9 and 7 which resulted in the CN loss on chromosome 9, and the nested deletion present on chromosome 9, (**B**) shows the enlarge view of nested deletion having the CN state of approximately 0.5 surrounded by CN state of 1.3 which contain *CDKN2A* and *CDKN2B* genes. Green map shows the reference map and blue map shows the nested deletion detected in this case.

**Figure 6:**
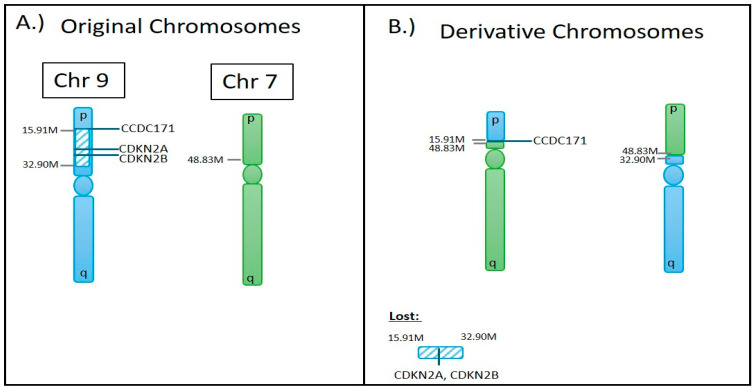
Graphic depiction of (**A**) the original chromosome involved in the translocation event between chromosome 7 and chromosome 9, (**B**) the resulting derivative chromosomes, including the lost section of chromosome 9 that encompasses *CDKN2A* and *CDKN2B* for the glioma case examined by CMA in Figure 4 and OGM in Figure 5.

In one case, OGM clearly defined an SV event that was detected with microarray analysis. In this case, a homozygous deletion of the cyclin-dependent kinase inhibitor 2A (*CDKN2A)* and cyclin-dependent kinase inhibitor 2B (*CDKN2B)* genes was detected in the microarray, as shown in Figure 4. OGM not only detected this event (Figure 5A) but also provided a more detailed insight into how the homozygous deletion happened. In this event, the microarray showed the copy number loss in the region p21.3 on chromosome 9 with a nested deletion, resulting in the biallelic loss of both copies of *CDKN2A* and *CDKN2B* genes. In contrast, OGM analysis uncovered an unbalanced translocation between chromosomes 7 and 9, resulting in a loss of regions between 15.91 M and 32.90 M on chromosome 9, with no loss or gain on chromosome 7 (Figure 5A and Figure 6A). There is trisomy of chromosome 7, which is an independent event. The resulting derivative chromosomes are shown in Figure 6B. This translocation ended up with the monoallelic loss of both *CDKN2A* and *CDKN2B* genes. But the second allele of chromosome 9 was not involved in this translocation event, as confirmed by a secondary/nested deletion on chromosome 9, leading to loss of *CDKN2A* and *CDKN2B* genes (Figure 5B).

### 3.4. OGM Detected the Cryptic SV Events

In addition to identifying the previously characterized SVs, OGM also detected some additional cryptic SV events (Supplementary File Table S2). Some of the examples are presented here.

#### 3.4.1. A Complex Translocation Event

**Figure 7:**
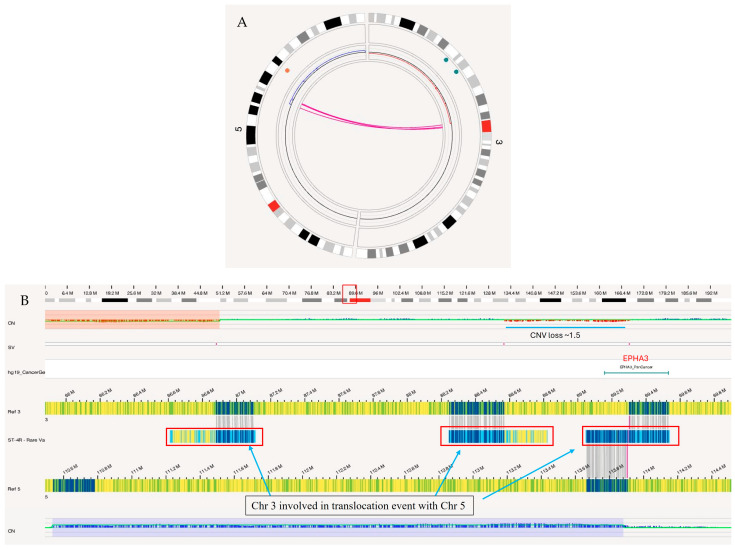
An unbalanced, complex translocation was detected in glioma case. (**A**) Circos plot showing the loss of p-arm on chromosome 3 and a copy number gain on chromosome 5 in SV track. (**B**) Enlarged view of the SV track showing three breakpoints on chromosome 3 involved in the translocation with chromosome 5, labeled with red rectangular boxes. One breakpoint at position 89.23 M on chromosome 3 results in the disruption of the *EPHA3* gene. In CN track, red is loss of copy number state and blue is copy number gain.

**Figure 8:**
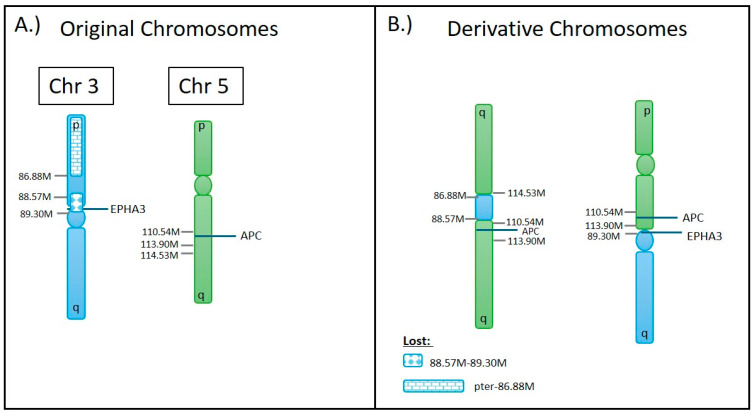
Graphic depiction of (**A**) the original chromosomes involved, (**B**) the movement of genetic material between chromosome 3 and chromosome 5 in the translocation identified by OGM. This translocation results in a loss of genetic material on 3p, a disruption of *EPHA3* on chromosome 3, a duplication on chromosome 5q (110.54–113.90 M), and a gain of chromosome 5q from 5q22.3/114.5 to the q-terminus.

OGM identified a complex translocation event t(3,5)(p12.1;q22.1) in another glioma case in which the portion of p-arm of chromosome 3 was lost, and a copy number gain was observed on chromosome 5, as shown in Figure 7. This event involved three translocation breakpoints, resulting in the derivative chromosome depicted in Figure 8. The first translocation breakpoint on chromosome 3 at ~86.88 M joins with q-arm of chromosome 5 at 114.3 M, causing a loss of the p-arm of chromosome 3. The second breakpoint on chromosome 3 at 88.57 M joins to a second copy of the q-arm of chromosome 5 at 110.54 M. This results in a derivative chromosome containing a portion of the p-arm of chromosome 3 (86.88 M to 88.57 M) flanked between two q-arms of chromosome 5, as shown in 8B. The presence of two 5q-arms creates a duplication from 5q22.3 to the q-terminus. Due to the non-homologous breaking of the two q-arms of chromosome 5, a section slightly upstream (110.54–113.90 M) has been duplicated and was present on both derivative chromosomes, as shown in Figure 8B. The end result is a duplication of the adenomatous polyposis coli (*APC*), a tumor suppressor gene. The third breakpoint results in the fusion of the q-arm of chromosome 3 with a single p-arm of chromosome 5 at 89.3 M and 113.9 M, respectively. As a result of this event, Ephrin type-A receptor 3 (*EPHA3*) on chromosome 3 was disrupted. The second half of the gene has been attached to chromosome 5, while the first half was lost due to a small segmental loss involved in the translocation of a small section of chromosome 3 (88.57–89.30 M) between two breakpoints on chromosome 3.

#### 3.4.2. Three-Way Translocation Event

**Figure 9:**
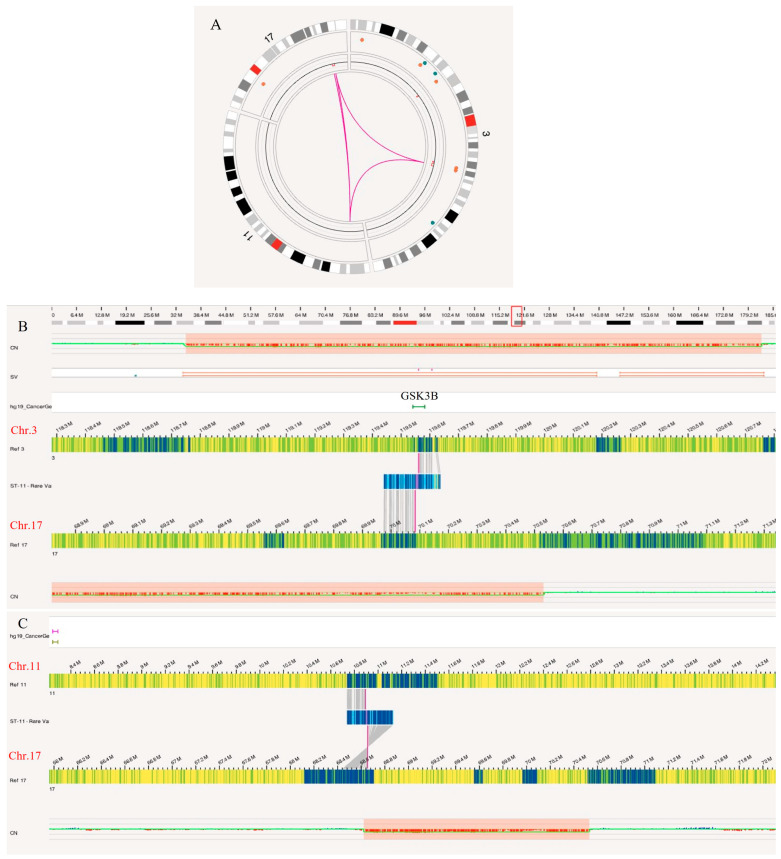
A three-way translocation between chromosomes 3, 11, and 17, as revealed by OGM in glioma case. (**A**) Circos plot of the translocation event, (**B**) enlarged SV view of a breakpoint between chromosome 3 and chromosome 17 involving gene *GSK3B*, and (**C**) enlarged SV view of a breakpoint between chromosome 11 and the inverted chromosome 17.

**Figure 10:**
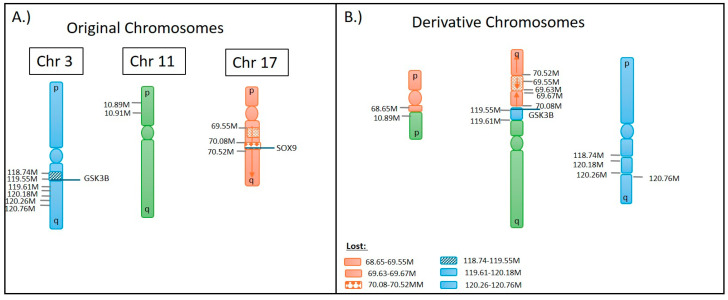
(**A**) Graphic depiction of the original chromosomes involved in the 3-way translocations and the original location of the genes that are affected by the translocation. (**B**) The derivative chromosomes identified by OGM and formed as a result of the 3-way translocation.

OGM identified a three-way translocation t(17,3,11)(q24.3;q13.33;p15.3), which was not revealed previously by CMA analysis (Figure 9). In this complex translocation, the q24.3 region of chromosome 17 attached to an inverted p-arm of chromosome 11 at p15.3, bringing the gene *ZBED5-AS1* in contact with q24.3 on chromosome 17 in a region with no genes present (68.65 M). A portion of chromosome 17, including the pan-cancer gene *SOX9,* was deleted at q24.3 and q25.1 due to the translocation. The remaining q-arm of chromosome 17 was inverted, partially deleted, and reattached upstream from 70.08 M to an 81 kb section of chromosome 3 (119.55–119.63 M). The breakpoint on the other side of the 81 kb section of chromosome 3 is the remaining chromosome 11 with no immediate genes involved in the breakpoint from either chromosome. The 81 kb section of chromosome 3 (119.55–119.63 M) involved in the translocation was accompanied by flanking deletions identified by OGM, spanning the region of 118.74 M to 120.76 M, that involve many genes, including *GSK3B*, a pan-cancer gene. The derivative chromosomes are shown in Figure 10B.

### 3.5. OGM Detected a Balanced Translocation Event

**Figure 11:**
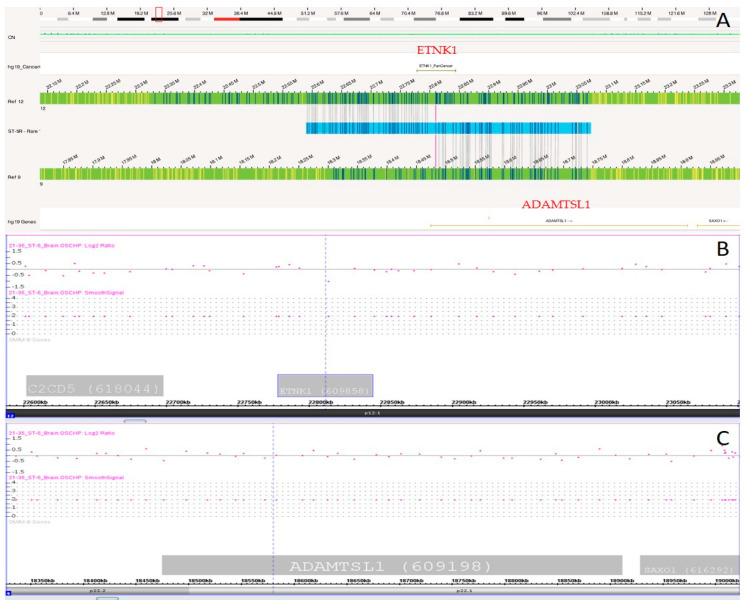
(**A**) OGM showing the balanced translocation event in which gene fusion was observed between *ETNK1* and *ADAMTSL1* gene. (**B**,**C**) No change in either of gene was observed in the CMA analysis.

OGM analysis detected a balanced translocation SV event in another case between chromosome 12 and 9, in which the fusion of *ETNK1*, a pan-cancer gene, and *ADAMTSL1* was observed in this event, as shown in Figure 11A. In this balanced translocation event, no gain or loss of a chromosome segment was observed on either chromosome, and fusion of these two genes was observed. In the CMA analysis, no change in the copy number state of either gene was observed, as shown in Figure 11B,C.

## 4. Discussion

The 2021 World Health Organization (WHO-2021) guidelines for gliomas emphasize the importance of precise molecular characterization for accurate diagnosis, prognosis, and treatment planning. Optical Genome Mapping (OGM) is revolutionizing the clinical practice of cytogenomic characterization, currently obtained through a combination of technologies such as karyotyping, FISH, and CMA. Recent reports, including those from our lab, have demonstrated the utility of OGM in the detection of clinically significant cytogenomic abnormalities in hematological malignancies and constitutional cases [11,12]. We show here that OGM analysis of gliomas can provide a comprehensive picture of the whole genome, enabling detailed analysis of different SVs, unraveling complex/cryptic rearrangements, and reliably identifying translocation events in a single step, potentially decreasing the turnaround time for the start of tailored treatment.

OGM data were found to be concordant with CMA in identifying previously characterized SVs and CNVs. In addition, OGM identified additional clinically relevant aberrations and demonstrated higher resolution and sensitivity in accurately defining different SV events. While CMA can detect CNVs as small as 50–100 kb, OGM can detect SVs down to 500 bp. Furthermore, CMA cannot detect translocations, inversions, smaller imbalances, or low-level mosaicism.

Chromothripsis is a catastrophic event in which chromosomal segments are shattered, reshuffled, and randomly stitched together, producing complex derivative chromosomes. Ramos-Campoy et al. described that OGM provides a more detailed description of this event than microarray in chronic lymphocytic leukemia (CLL) cases, due to better characterization of translocations, distinct rearrangement patterns, and breakpoints [13]. Similarly, in our study, CMA presented the results of a particular case as a simple deletion of a region of chromosome 9 involving *CDKN2A* and *CDKN2B*. OGM, however, provided a clear presentation of the SV events involved in an unbalanced translocation coupled with a secondary deletion. In this event, the deletion on one allele was supported by a translocation, while the deletion on the other allele was due to a secondary deletion on chromosome 9. OGM accurately identified the breakpoints involved in these events, supporting both gene deletions, whereas microarray could only define the presence of the deletion without identifying the translocation pattern. In a similar study, microarray failed to detect unbalanced chromosome abnormalities in six postnatal cases, which were detected by chromosome analysis [14]. These cases exhibited low-level mosaicism, ranging between 2% and 7.5%, below CMA’s detection limit. OGM, with a detection capability down to 5%, demonstrates higher resolution in detecting low-level mosaic events [7].

OGM also showed the disruption of the *EPHA3* gene, which is implicated in gliomas and overexpressed in 60% of cases, making it a target for glioma therapy [15]. Although *EPHA3* is associated with tumor initiation and infiltration in gliomas, no correlation between its disruption/rearrangement and gliomas has been reported in the literature. Additionally, in a three-way translocation, the *GSK3* gene was found to be disrupted. This gene is involved in the differentiation of malignant glial cells via critical downstream signaling protein pathways. This gene was found to be disrupted and deleted by OGM analysis, but only copy number change/deletion of *GSK3* was observed in CMA. OGM provided the better resolution of an SV event here, with correct position of a translocated gene and its possible interactions with other genes, when compared with CMA. OGM detected a balanced translocation event which was not detected in the CMA analysis. This event leads to a gene fusion between the *ETNK1* and *ADAMTSL1* genes. *ADAMTSL1* is involved in promoting the progression of gliomas [16] and is a potential prognostic biomarker for malignant invasion in glioma.

Microarray assays are limited to detecting genome-wide copy number changes, and their capacity depends on the number of probes used. In contrast, OGM can detect various types of SVs genome-wide in a single run, providing detailed information about translocated partners, inverted regions, and gene fusions to help understand an SV event, which can be helpful for the sub-typing of gliomas. This capability is critical for cancer diagnosis and will help in making better diagnostic and therapeutic decisions, resulting in better patient outcomes.

## 5. Conclusions

This OGM approach helps in the detection of all classes of SVs in a single assay and generates clinically actionable high-resolution genomic data. OGM can be incorporated in the laboratory workflow and will facilitate precise diagnosis and better prognostication of gliomas. This would help with making better therapeutic decisions, establishing new insight for this class of solid tumors by uncovering additional clinically relevant SVs. 

## Data Availability

The original contributions presented in the study are included in the article/supplementary material, further inquiries can be directed to the corresponding author/s.

## Supplementary Materials

The following supporting information can be downloaded at: https://www.mdpi.com/article/10.3390/biomedicines12081659/s1, Supplementary Table S1: Showing the quality control metrics of the tumor samples for OGM, Supplementary Table S2: Showing the findings of OGM in different tumor samples.

## References

[1] Cancer Stat Facts: Brain and Other Nervous System Cancer. https://seer.cancer.gov/statfacts/html/brain.html.

[2] Liang J., Lv X., Lu C., Ye X., Chen X., Fu J., Luo C., Zhao Y. (2020). Prognostic factors of patients with gliomas–an analysis on 335 patients with glioblastoma and other forms of gliomas. BMC Cancer.

[3] McNeill K.A. (2016). Epidemiology of brain tumors. Neurol. Clin..

[4] Louis D.N., Perry A., Wesseling P., Brat D.J., Cree I.A., Figarella-Branger D., Hawkins C., Ng H.K., Pfister S.M., Reifenberger G. (2021). The 2021 WHO classification of tumors of the central nervous system: A summary. Neuro-Oncology.

[5] Mantere T., Neveling K., Pebrel-Richard C., Benoist M., van der Zande G., Kater-Baats E., Baatout I., van Beek R., Yammine T., Oorsprong M. (2021). Optical genome mapping enables constitutional chromosomal aberration detection. Am. J. Hum. Genet..

[6] Garcia-Heras J. (2021). Optical Genome Mapping: A Revolutionary Tool for “Next Generation Cytogenomics Analysis” with a Broad Range of Diagnostic Applications in Human Diseases. J. Assoc. Genet. Technol..

[7] Sahajpal N.S., Mondal A.K., Tvrdik T., Hauenstein J., Shi H., Deeb K.K., Saxe D., Hastie A.R., Chaubey A., Savage N.M. (2022). Clinical validation and diagnostic utility of optical genome mapping for enhanced cytogenomic analysis of hematological neoplasms. J. Mol. Diagn..

[8] Levy B., Baughn L.B., Akkari Y., Chartrand S., LaBarge B., Claxton D., Lennon P.A., Cujar C., Kolhe R., Kroeger K. (2023). Optical genome mapping in acute myeloid leukemia: A multicenter evaluation. Blood Adv..

[9] Sahajpal N.S., Barseghyan H., Kolhe R., Hastie A., Chaubey A. (2021). Optical Genome Mapping as a Next-Generation Cytogenomic Tool for Detection of Structural and Copy Number Variations for Prenatal Genomic Analyses. Genes.

[10] SP Tissue and Tumor DNA Isolation Kit. https://bionano.com/sp-tissue-and-tumor-dna-isolation-kit.

[11] Neveling K., Mantere T., Vermeulen S., Oorsprong M., van Beek R., Kater-Baats E., Pauper M., van der Zande G., Smeets D., Weghuis D.O. (2021). Next-generation cytogenetics: Comprehensive assessment of 52 hematological malignancy genomes by optical genome mapping. Am. J. Hum. Genet..

[12] Xie M., Zheng Z.J., Zhou Y., Zhang Y.X., Li Q., Tian L.Y., Cao J., Xu Y.T., Ren J., Yu Q. (2024). Prospective Investigation of Optical Genome Mapping for Prenatal Genetic Diagnosis. Clin. Chem..

[13] Ramos-Campoy S., Puiggros A., Kamaso J., Beà S., Bougeon S., Larráyoz M.J., Costa D., Parker H., Rigolin G.M., Blanco M.L. (2022). TP53 abnormalities are underlying the poor outcome associated with chromothripsis in chronic lymphocytic leukemia patients with complex karyotype. Cancers.

[14] Bi W., Borgan C., Pursley A.N., Hixson P., Shaw C.A., Bacino C.A., Lalani S.R., Patel A., Stankiewicz P., Lupski J.R. (2013). Comparison of chromosome analysis and chromosomal microarray analysis: What is the value of chromosome analysis in today’s genomic array era. Genet. Med..

[15] Ferluga S., Tomé C.M.L., Herpai D.M., D'Agostino R., Debinski W. (2016). Simultaneous targeting of Eph receptors in glioblastoma. Oncotarget.

[16] Wang S., Zhang J., Wang K., Zhao Y., Liu D. (2023). ADAMTS1 as potential prognostic biomarker promotes malignant invasion of glioma. Int. J. Clin. Oncol..

